# The Impact of the COVID-19 Pandemic and Lockdown on Mild Cognitive Impairment, Alzheimer's Disease and Dementia With Lewy Bodies in China: A 1-Year Follow-Up Study

**DOI:** 10.3389/fpsyt.2021.711658

**Published:** 2021-07-28

**Authors:** Zhi-Chao Chen, Shuai Liu, Jinghuan Gan, Lingyun Ma, Xiaoshan Du, Han Zhu, Jiuyan Han, Junying Xu, Hao Wu, Min Fei, Yuchao Dou, Yaqi Yang, Peng Deng, Xiao-Dan Wang, Yong Ji

**Affiliations:** ^1^Department of Neurology, China National Clinical Research Center for Neurological Diseases, Beijing Tiantan Hospital, Capital Medical University, Beijing, China; ^2^Tianjin Key Laboratory of Cerebrovascular and of Neurodegenerative Diseases, Department of Neurology, Tianjin Dementia Institute, Tianjin Huanhu Hospital, Tianjin, China; ^3^Graduate School of Tianjin Medical University, Tianjin, China; ^4^Department of Neurology, Tianjin Baodi People's Hospital, Tianjin, China; ^5^Department of Neurology, Yuncheng Central Hospital, Shanxi, China; ^6^Tianjin Umbilical Cord Blood Hematopoietic Stem Cell Bank, Tianjin, China

**Keywords:** COVID-19, social isolation, physical activities, Alzheiemer's disease, dementia with Lewy bodies

## Abstract

**Background:** While the lockdown strategies taken by many countries effectively limited the spread of COVID-19, those were thought to have a negative impact on older people. This study aimed to investigate the impact of lockdown on cognitive function and neuropsychiatric symptoms over a 1-year follow-up period in patients with mild cognitive impairment (MCI), Alzheimer's disease (AD) and dementia with Lewy bodies (DLB).

**Methods:** We enrolled consecutive patients with MCI, probable AD or DLB who were receiving outpatient memory care before the COVID-19 pandemic and followed-up with them after 1 year by face-to-face during the COVID-19 pandemic to assess changes in physical activity, social contact, cognitive function and neuropsychiatric symptoms (NPS).

**Results:** Total 105 probable AD, 50 MCI and 22 probable DLB patients were included and completed the 1-year follow-up between October 31 and November 30, 2020. Among the respondents, 42% of MCI, 54.3% of AD and 72.7% of DLB patients had a decline in MMSE scores and 54.4% of DLB patients had worsening Neuropsychiatric inventory (NPI) scores. Patients with DLB showed a more rapid decline of MMSE than those with AD. Diminished physical activity and social contact might have hastened the deterioration of cognition and the worsening of NPS.

**Conclusion:** Social isolation and physical inactivity even after strict lockdown for at least 6 months were correlated with accelerated decline of cognitive function and NPS in patients with AD and DLB.

## Introduction

An outbreak of coronavirus disease 2019 (COVID-19), caused by severe acute respiratory syndrome coronavirus 2 (SARS-CoV-2), began in December 2019 in Wuhan, Hubei, China, and rapidly spread across the globe within approximately 3 months (1). The Chinese government acted immediately and put the whole country into lockdown starting from 23 January 2020 to 23 February 2020. From February to April, citizens were still asked to stay at home and limit outdoor activities. The World Health Organization declared the COVID-19 pandemic on 11 March 2020.

Social distancing is practiced by canceling events and gatherings, closing public places, working at home, avoiding physical contact and implementing travel restrictions. In China, every citizen was given a permission card and only allowed to leave home every second day for a maximum of 30 min. As such, outside physical activities were extremely limited. In consideration of the elevated risk of infection and death in the elderly, experts especially reminded them to reduce outdoor activities (2).

Patients with cognitive impairment are mostly aged over 60 years, and their physical and mental health were directly and indirectly affected by the COVID-19 pandemic. As is well-known, physical inactivity is a modifiable risk factor for Alzheimer's disease (AD). Preliminary data indicate that physical activity (PA) levels decreased among older adults by 26.5% during the pandemic (3). Researchers may pay more attention to the impact of worsening cognitive function. Several studies have focused on neuropsychiatric symptoms (NPS) and mental health in elderly people and dementia patients (4, 5). They showed that the COVID-19 pandemic had a wide negative impact on the mental well-being of older adults with and without dementia. However, most of those studies are case reports or cross-sectional studies with a small sample size. So far, no studies have followed dementia patients for at least 1 year during the COVID-19 pandemic and did not discuss the different change between different cognitive impairment subtypes. Mild cognitive impairment (MCI) usually has memory loss, but it is different from AD for it does not typically affect a person's ability to complete daily task. Some people suffered dementia with Lewy bodies (DLB) have both cognitive impairment and parkinsonism. And the prevalence of hallucination in DLB was higher than AD. The aim of our study was to investigate changes in cognitive function and NPS during the first year of the COVID-19 pandemic in patients with mild cognitive impairment, AD and dementia with Lewy bodies (DLB) and to find the similarities or differences between different dementia types. In addition, we explored the predictive factors at baseline or during lockdown periods, especially in terms of physical activity and social contact, for the worsening or improvement of those symptoms. We supposed that the cognitive and neuropsychiatric symptoms decline faster in DLB and AD than in MCI. And the decline of cognition and neuropsychiatric symptoms in the dementia group may relate to the decrease of physical activity and social contact.

## Methods

### Design and Participants

**Figure 1:**
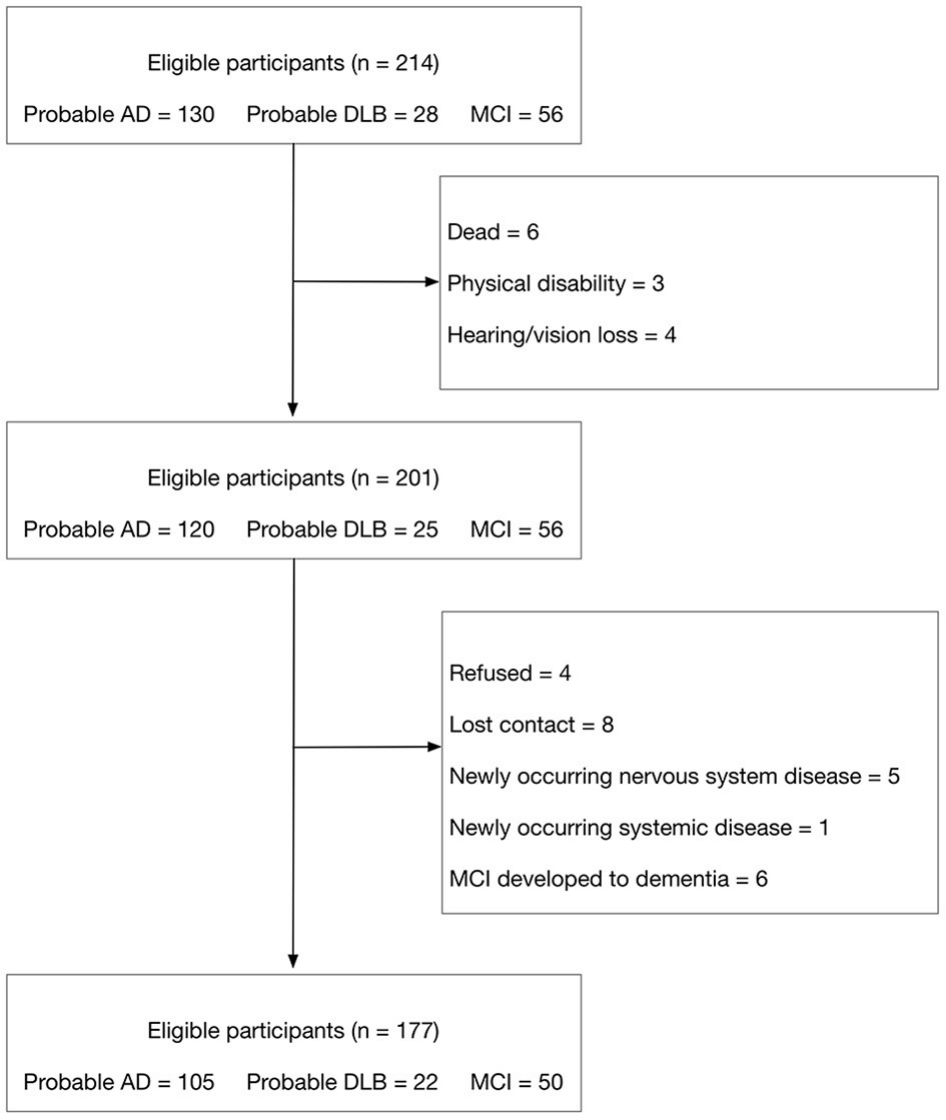
Flow chart for inclusion and exclusion.

This was a 1-year longitudinal and observational study, which was conducted at the memory clinic in Tianjin Huanhu Hospital. We consecutively recruited a total of 214 participants [probable Alzheimer's disease (AD) = 130, mild cognitive impairment (MCI) = 56, probable dementia with Lewy bodies (DLB) = 28] who underwent evaluation of cognitive function and NPS from 30 September 2019 to 31 December 2019, before the COVID-19 pandemic. The diagnoses of probable AD and MCI were based on the National Institute on Aging and Alzheimer's Association (NIA-AA) criteria (6), and the diagnosis of probable DLB was according to the fourth consensus report of the DLB consortium (7). Blood tests, neurological examination, neuroimaging (including CT scans or MRI), and positron emission computerized tomography (including FDG-PET and amyloid PET) if necessary, were performed to make the diagnosis. All clinical diagnoses of dementia were made by consensus agreement of at least two experienced neurologists. The exclusion criteria included severe loss of vision or hearing, physical disability, lost to follow-up, newly occurring delirium, strokes, and life-threatening illness. At the 1-year follow-up we reassessed the neurological examination etc. and recorrected the diagnosis, excluded the participants who developed into dementia in MCI group or recorrected diagnosis as other disease in DLB and AD groups. Finally, 177 participants (probable AD = 105, MCI = 50 and probable DLB = 22) completed all of the evaluations of cognition and NPS face to face (Figure 1). All the information except cognition assessment was all collected from the caregivers of participants.

### Assessment

Our study was designed to have two time points of observation. At baseline and 1-year follow-up, the neurologists recorded the clinical characteristics, lifestyle, medical history, disease history and medication use of the participants. Marital status was divided into married and unmarried and education level into low (completed 0 to 12 years) and high (13 years and above). Lifestyle behaviors include smoke and drink. A smoker was defined as an individual with a history of smoking ≥ 5 cigarettes per day for >2 years. An alcohol drinker was defined as an individual with a history of drinking an alcoholic beverage ≥ 1 time per week for >2 years. We set up a self-designed questionnaire with reference to a health survey (8), which combined questions on frequency, duration and intensity to form a summary index of weekly PA, weighted by intensity level. Social contact was measured by using a self-rated questionnaire reference to a cohort study that assessed the number and frequency of contact with relatives and friends (9). Baseline PA and social contact was evaluated before the COVID-19 pandemic, and 1 year later, neurologists interviewed the patients retrospectively to assess the change of PA and social contact during the January to April 2020 lockdown. Global cognition was measured with Mini-Mental Status Exam (MMSE) and Montreal Cognitive Assessment (MOCA). Neuropsychiatric inventory (NPI) was used to assess the frequency and degree of NPS and depression was evaluated by Hamilton depression rating (HAMD). Subjects with severe depression (HAMD > 30 scores) were excluded in our study. The capacity to perform activities of daily living (ADL) was assessed with the ADL scale and daytime sleepiness was assessed by Epworth sleepiness scale (ESS).

### Statistical Analysis

Clinical characteristics and disease history are described in means ± standard deviation for continuous variables and numbers (percentages) for categorical variables in three groups of gender, age, disease duration, clinical dementia rating (CDR) stage, education level, marital status, comorbidities and medication use. The normality of the distribution was analyzed using the Shapiro-Wilk test. Descriptive statistics were used to assess differences among MCI, probable DLB and AD groups by using an analysis of variance (ANOVA) followed by Bonferroni-corrected pairwise comparison test for continuous variables and by using chi-squared test, Pearson's test and Fisher's exact test for categorical variables. The comparison of means at baseline and 1 year was made by paired-samples Student's *t* test. The comparison between categorical variables was performed by using chi-squared, Pearson's test and Fisher's exact test. Analysis of correlation was made by using the Pearson's or Spearman's correlation, as appropriate. A multiple linear regression analysis was used to find possible risk factors for worsening MMSE and NPI scores in AD and DLB patients during the COVID 19 pandemic. Analysis of correlation was made by using the Pearson's *r* or Spearman's ρ, as appropriate. All of the data analyses were performed with SPSS Statistics 25.0.

This study was designed and conducted in accordance with the Declaration of Helsinki and written informed consent was signed by all participants.

## Results

Total 177 patients (probable AD = 105, MCI = 50, probable DLB = 22) finished the 1-year longitudinal follow-up study.

### Baseline Patient Characteristics in Three Groups

The baseline characteristics of the patients with MCI, AD and DLB are shown in Table 1. The age of the patients in the DLB group was significantly higher than that of the MCI group, but not significantly different from the AD group. Notably, the prevalence of sleep disturbance was much higher in DLB compared to AD and MCI. Utilization of acetylcholinesterase inhibitors and NMDA receptor inhibitors was higher in AD and DLB than in MCI, while there was no significant difference between AD and DLB. All three groups were similar in gender, education level, marital status, CDR stage and disease history. As for the scales, the MMSE, MOCA, and ADL scores were lower in AD and DLB than in MCI, but there were no significant differences between the AD and DLB groups. Patients with AD and DLB had higher NPI and HAMD scores than those with MCI, and depression and NPS were most severe in DLB.

**Table 1:** Baseline characteristics of patients with mild cognitive impairment (MCI), Alzheimer's disease (AD) and probable dementia with Lewy bodies (DLB).

**General characteristics**	**MCI (*n* = 50)**	**AD (*n* = 105)**	**DLB (*n* = 22)**	***P***
Age (years, mean ± SD)	68.7 ± 8.5	71.5 ± 8.1	74.0 ± 7.9^f^	0.030*
Gender, female (*n*, %)	29 (57.9%)	61 (58.0%)	10 (45.4%)	0.536
Disease duration (years)	2.8 ± 1.6	4.3 ± 2.4^b^	3.0 ± 1.6	<0.001***
Education level (*n*, %)				0.594
Low (0–12 years)	38 (76.0%)	87 (82.8%)	18 (81.8)	
High (≥13 years)	12 (24.0%)	18 (17.2%)	4 (18.2%)	
Marital status (*n*, %)				0.465
Married	38 (76.0%)	84 (80%)	15 (68.2%)	
Unmarried	12 (24.0%)	21 (20%)	7 (31.8%)	
CDR stage (*n*, %)				0.722
0.5	50 (100%)			
1		48 (45.7%)	8 (36.4%)	
2		32 (30.4%)	8 (36.4%)	
3		25 (23.9%)	6 (27.2%)	
Hypertension (*n*, %)	22 (44.0%)	41 (39.0%)	8 (36.3%)	0.782
Diabetes (*n*, %)	15 (30.0%)	15 (14.3%)	4 (18.1%)	0.067
Cardiovascular disease (*n*, %)	8 (16.0%)	9 (8.8%)	2 (9.0%)	0.364
Stroke (*n*, %)	6 (12.0%)	11 (10.5%)	1 (4.5%)	0.620
Sleep disturbance (*n*, %)	13 (26.0%)	34 (32.4%)	19 (86.4%)^fh^	<0.001***
Acetylcholinesterase inhibitors (*n*, %)	21 (42.0%)	69 (65.7%)^c^	16 (72.7%)^e^	0.008**
NMDA receptor inhibitors (*n*, %)	3 (6.0%)	24 (22.9%)^a^	5 (22.7%)^d^	0.032*
Physical activity (mean ± SD)	2.2 ± 2.6	4.0 ± 3.4^a^	2.5 ± 3.7	0.003**
Social contact (mean ± SD)	7.2 ± 2.8	5.7 ± 3.6^a^	4.9 ± 3.4^d^	0.007**
MMSE (mean ± SD)	24.5 ± 4.8	13.0 ± 7.0^c^	13.7 ± 7.0^f^	<0.001***
MOCA (mean ± SD)	20.4 ± 4.4	8.8 ± 5.7^c^	9.9 ± 5.9^f^	<0.001***
NPI (mean ± SD)	2.7 ± 5.0	9.6 ± 11.3^c^	18.6 ± 14.4^fh^	<0.001***
ADL (mean ± SD)	20.7 ± 1.1	34.7 ± 14.3^c^	36.1 ± 14.8^f^	<0.001***
HAMD (mean ± SD)	2.2 ± 3.9	4.2 ± 4.2	9.3 ± 8.0 ^fh^	<0.001***
ESS (mean ± SD)	1.0 ± 1.7	2.6 ± 4.0^a^	5.5 ± 4.8 ^fg^	<0.001***

MCI vs. AD vs. DLB *P < 0.05, **P < 0.01, ***P < 0.001.

*MCI vs. AD ^a^P < 0.05, ^b^P < 0.01, ^c^P < 0.001*.

*MCI vs. DLB ^d^ P < 0.05, ^e^P < 0.01, ^f^P < 0.001*.

*AD vs. DLB ^g^P < 0.01, ^h^P < 0.001*.

*CDR, clinical dementia rating; MMSE, mini-mental status; MOCA, Montreal cognitive assessment; NPI neuropsychiatric inventory; ADL, activities of daily living; HAMD, Hamilton depression scale; ESS, Epworth sleepiness scale*.

### Changes in Three Groups Between Baseline and 1-Year Follow-Up

**Figure 2:**
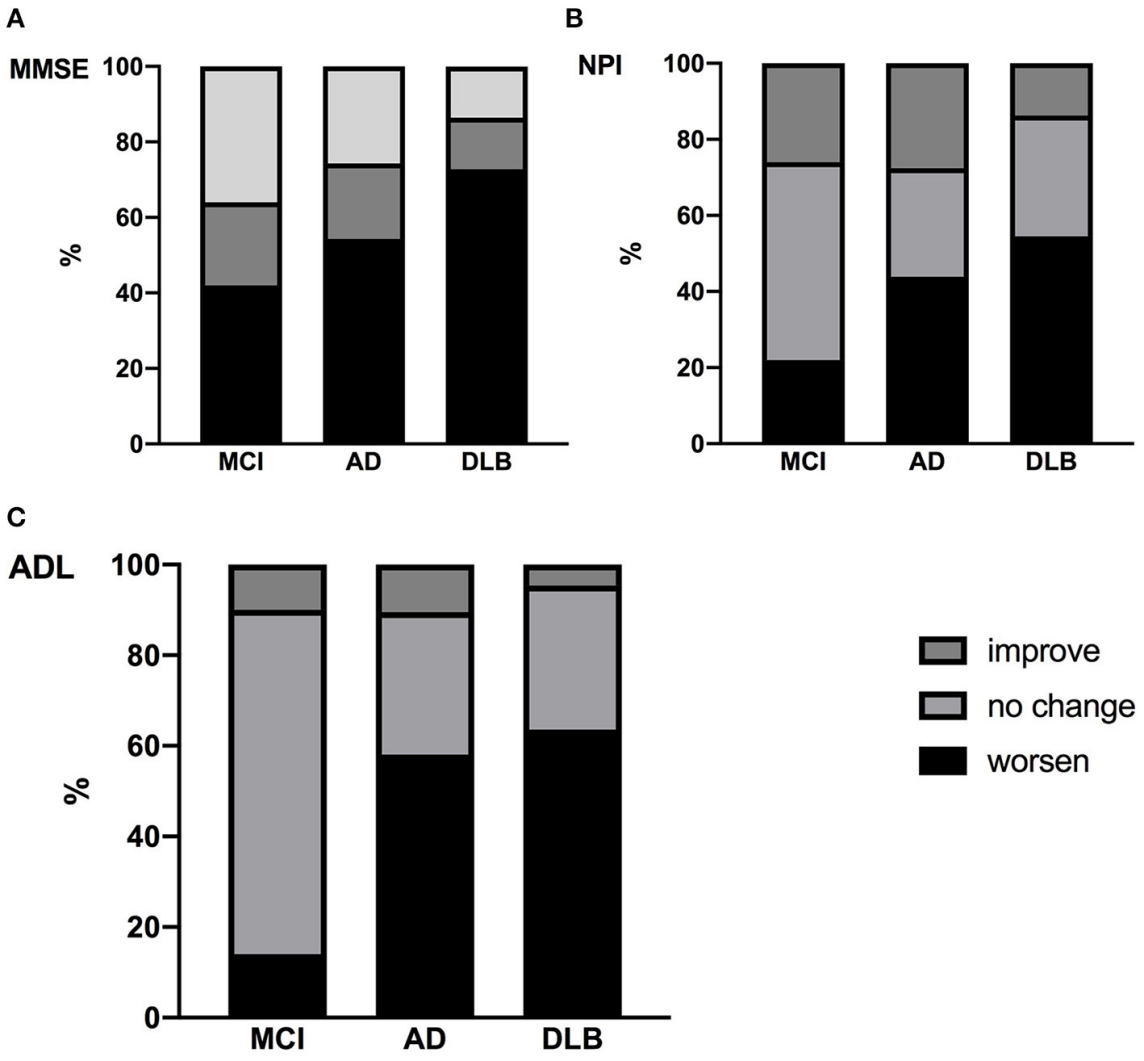
Comparison of changes in Mini-Mental Status Exam (MMSE) **(A)**, neuropsychiatric inventory (NPI) **(B)** and activities of daily living (ADL) **(C)** in three groups.

As shown in Figure 2, MMSE scores declined in 42% of MCI, 54.3% of AD and 72.7% of DLB patients during the COVID-19 pandemic, but the difference was significant only between MCI and DLB. Also, there was an increasing trend from MCI to AD and DLB in the rate of deterioration of NPI and ADL.

Table 2 shows the decline of PA, social contact, cognition and neuropsychiatric symptoms in patients with MCI, AD and DLB during the COVID-19 pandemic compared to the pre-pandemic situation. PA and social contact were both significantly decreased during the pandemic period in all three groups. Cognitive function, evaluated by MMSE and MOCA, and NPS evaluated by NPI and HAMD, did not show obvious change in patients with MCI, but in patients with AD, MMSE, MOCA, NPI, ADL, HAMD, and ESS were markedly worsened during the COVID-19 pandemic. DLB patients also had lower MMSE and MOCA scores compared to the pre-pandemic baseline scores. However, the NPI, HAMD and ESS scores worsened or improved only slightly and without significant differences in the DLB group.

**Table 2:** Comparison of baseline and 1-year follow-up data for patients with mild cognitive impairment (MCI), Alzheimer's disease (AD) and probable dementia with Lewy bodies (DLB).

	**MCI (** ***n*** **=** **50)**				**AD (** ***n*** **=** **105)**				**DLB (** ***n*** **=** **22)**			
	**Baseline**	**1 year later**	**Change**	***P***	**Baseline**	**1year later**	**Change**	***P***	**Baseline**	**1year later**	**Change**	***P***
Physical activity	4.0 ± 3.4	1.7 ± 2.1	1.5 ± 2.9	<0.001***	3.2 ± 3.1	1.7 ± 2.1	1.5 ± 2.2	<0.001***	2.5 ± 3.7	1.1 ± 2.0	1.4 ± 2.2	0.006**
Social contact	7.2 ± 2.8	6.4 ± 3.3	0.9 ± 2.4	0.014*	5.7 ± 3.6	4.3 ± 3.5	1.3 ± 3.2	<0.001***	4.9 ± 3.4	3.0 ± 3.4	1.9 ± 2.5	0.003**
MMSE	24.5 ± 4.8	24.0 ± 5.8	0.4 ± 3.6	0.298	13.0 ± 7.0	11.4 ± 7.4	1.6 ± 3.4	<0.001***	13.7 ± 7.0	10 ± 6.9	3.6 ± 5.5 ^c^	0.002**
MOCA	20.4 ± 4.4	20.1 ± 5.5	0.2 ± 3.4	0.560	8.8 ± 5.7	7.8 ± 5.9	1.0 ± 3.2 ^a^	0.001**	9.9 ± 5.9	7.3 ± 5.4	2.5 ± 5.0 ^c^	0.007**
NPI	2.7 ± 5.0	2.8 ± 5.2	0.1 ± 2.6	0.710	9.7 ± 11.2	12.2 ± 13.5	2.7 ± 8.2	0.008**	18.6 ± 14.4	20.9 ± 14.8	2.5 ± 10.4	0.139
ADL	20.7 ± 1.1	21.3 ± 2.4	0.6 ± 2.2	0.127	34.7 ± 14.3	41.1 ± 17.8	6.4 ± 9.8 ^b^	<0.001***	36.1 ± 14.8	44.7 ± 20.3	8.6 ± 12.2 ^c^	0.002**
HAMD	2.2 ± 3.9	2.9 ± 5.3	0.7 ± 4.4	0.726	4.2 ± 4.4	4.9 ± 5.1	0.8 ± 2.6	0.002**	9.3 ± 8.0	9.2 ± 6.9	−0.1 ± 5.9	0.665
ESS	1.0 ± 1.7	1.3 ± 2.4	0.2 ± 1.1	0.107	2.6 ± 4.0	3.4 ± 5.1	0.8 ± 2.2	<0.001***	5.5 ± 4.8	6.4 ± 6.4	1.3 ± 3.7	0.201

Baseline mean compared with 1-year follow-up, *P < 0.05, **P < 0.01, ***P < 0.001.

*Changes of the evaluated scores compared between MCI and AD, ^a^P < 0.05, ^b^P < 0.001; changes of the evaluated scores compared between MCI and DLB, ^c^P < 0.01. MMSE, mini mental status exam; MOCA, Montreal cognitive assessment; NPI neuropsychiatric inventory; ADL, activities of daily living; HAMD, Hamilton depression scale; ESS, Epworth sleepiness scale*.

Table 2 also shows the mean values and SD of the changes in PA, social contact, MMSE, MOCA, NPI, ADL, HAMD and ESS for the three groups. The decline of PA and social contact seemed similar among the three groups. We found significant differences in cognition between DLB and MCI but not between MCI and AD or AD and DLB. The MMSE score declined by 3.6 points and the MOCA by 2.5 points in the DLB patients during COVID-19. Corresponding to the cognitive decline were various degrees of decline in ADL for patients in the AD (6.4 ± 9.8) and DLB (8.6 ± 12.2) groups. The drop in ADL in AD and DLB was significantly greater than in MCI. We additionally noticed that the degree of deterioration of NPI, HAMD, and ESS was not significantly different among the three groups.

### Predictors of Cognitive and Neuropsychiatric Decline in Alzheimer's Disease and Probable Dementia With Lewy Bodies

We checked for the association between cognitive decline (MMSE scores), worsening of neuropsychiatric symptoms (NPI scores) and hypothesized risk factors including baseline characteristics, lifestyle, sleep disturbance and baseline MMSE, NPI, HAMD, and ESS in patients with AD and DLB (Table 3). We found that the decline in PA (*r* = 0.274, *P* = 0.005) and the presence of sleep disturbance (*r* = 0.208, *P* = 0.033) were positively correlated to declining MMSE scores in AD. Several factor s were also correlated with NPI in AD, including social contact (*r* = 0.273, *P* = 0.005), sleep disturbance (*r* = 0.279, *P* = 0.004), baseline MMSE (*r* = −0.274, *P* = 0.005) and ESS (*r* = 0.298, *P* = 0.002). In DLB patients, there was no correlation between declining MMSE scores and other hypothesized factors, and the change in social contact (*r* = 0.496, *P* = 0.029) was the only factor with a significant positive correlation to the worsening of NPI scores.

**Table 3:** Correlation between cognitive and neuropsychiatric decline and hypothesized related factors in Alzheimer's disease (AD) and probable dementia with Lewy bodies (DLB).

	**AD**				**DLB**			
	**MMSE worse**		**NPI worse**		**MMSE worse**		**NPI worse**	
	***r***	***P***	***r***	***P***	***r***	***P***	***r***	***P***
Age (baseline)	−0.030	0.760	0.077	0.434	0.147	0.514	0.141	0.531
Gender	0.001	0.992	0.061	0.535	−0.101	0.654	0.044	0.846
Disease duration (baseline)	−0.09	0.924	−0.043	0.664	−0.383	0.079	−0.149	0.507
Marital status (baseline)	0.170	0.083	0.034	0.729	−0.232	0.299	−0.251	0.260
Education level	0.053	0.592	−0.134	0.172	0.205	−0.359	0.350	0.110
Physical activity (change)	0.274	0.005**	0.040	0.685	−0.142	0.527	0.299	0.177
Social contact (change)	0.069	0.485	0.273	0.005**	0.136	0.547	0.496	0.029*
Sleep disturbance (baseline)	0.208	0.033*	0.279	0.004**	0.210	0.349	0.001	1.000
MMSE (baseline)	0.002	0.987	−0.274	0.005**	0.382	0.080	0.182	0.417
NPI (baseline)	0.137	0.163	−0.090	0.359	−0.120	0.594	−0.333	0.131
HAMD (baseline)	0.125	0.203	0.175	0.075	−0.032	0.889	−0.341	0.121
ESS (baseline)	0.124	0.209	0.298	0.002**	−0.014	0.953	0.058	0.804

*P < 0.05, **P < 0.01; MMSE, mini-mental status exam; NPI neuropsychiatric inventory; HAMD, Hamilton depression scale; ESS, Epworth sleepiness scale.

Multiple linear regression analysis was used to find predictors of cognitive decline and worsening NPS in AD (Table 4). After adjusting for age, gender, disease duration, sleep disturbance and baseline MMSE, NPI, HAMD, and ESS, the results indicated that lower PA predicted a more rapid decline of MMSE scores in AD. It seemed that the decline of social contact along with sleep disturbance with high ESS scores at baseline could be blamed for worsening of NPI scores in AD. Considering the small sample size in DLB, we only adjusted for age, gender, physical activity and sleep disturbance in the multiple linear regression analysis of MMSE and NPI changes (Table 5). Less social contact was a risk factor for worsening of both MMSE and NPI scores during the COVID-19 pandemic.

**Table 4:** Multiple linear regression analysis of cognitive and neuropsychiatry changes in Alzheimer's Disease.

	**MMSE**		**NPI**	
	**Beta (SD)**	***P***	**Beta (SD)**	***P***
Age	−0.065	0.511	0.042	0.655
Gender	−0.016	0.872	0.130	0.161
Disease duration	−0.002	0.986	−0.014	0.880
Physical activity (change)	0.307	0.003**	0.049	0.609
Social contact (change)	−0.131	0.219	0.204	0.045*
Sleep disturbance	0.157	0.127	0.228	0.021*
MMSE (baseline)	0.141	0.177	−0.137	0.168
NPI (baseline)	0.131	0.297	−0.194	0.105
HAMD (baseline)	0.115	0.410	0.051	0.697
ESS (baseline)	0.085	0.427	0.253	0.014*
Adjusted R^2^		0.101		0.193
*P*-value of F		0.027*		0.001**

*P < 0.05, **P < 0.01; MMSE, mini-mental status exam; NPI, neuropsychiatric inventory; HAMD, Hamilton depression scale; ESS, Epworth sleepiness scale.

**Table 5:** Multiple linear regression analysis of cognitive and neuropsychiatry changes in dementia with Lewy bodies.

	**MMSE**		**NPI**	
	**Beta**	***P***	**Beta**	***P***
Age	0.404	0.125	0.415	0.093
Gender	−0.192	0.499	−0.060	0.814
Physical activity (change)	−0.132	0.540	0.251	0.217
Social contact (change)	0.491	0.034*	0.552	0.013*
Sleep disturbance	0.078	0.716	0.044	0.824
Adjusted R^2^		0.176		0.288
*P*-value of F		0.042*		0.049*

*P < 0.05; MMSE, mini-mental status exam; NPI, neuropsychiatric inventory.

## Discussion

This study is the first longitudinal 1-year follow-up study of dementia during the COVID-19 pandemic. The study included patients with MCI, AD, and DLB who experienced lockdown for about 4 months during the pandemic, and we investigated the influence of lockdown and stay-at-home mandates on the decline of cognitive and neuropsychiatric function. The study found that patients in the DLB group had the highest proportion of decline in cognitive and neuropsychiatric function. Moreover, the sudden decrease in PA during lockdown predicted the decline of MMSE scores at the 1-year follow-up in AD patients, and the decrease in social contact appeared to be a risk factor for the worsening of NPS.

Our longitudinal study showed different degrees of deterioration of cognitive function, NPS and ADL after the lockdown in AD and DLB. At the 1-year follow-up, we found that patients with AD had an average cognitive decline (MMSE) of 1.6 points and those with DLB lost 3.6 points during the COVID-19 pandemic. The drop of MMSE scores in AD was similar to the rate of yearly decline (~1.6 points per year) in previous studies without lockdown. However, in DLB, the mean annual decline in MMSE scores in a long-term multicenter cohort was 2.1 points (10). A prior single-center study with 67 patients who were followed for up to 5 years also showed a more rapid decline, by ~1 point per year, in DLB compared to AD (11). In our study, the MMSE scores in patients DLB dropped more than twice as fast as those in patients with AD during the first year of COVID-19. This could imply that during lockdown and stay-at-home mandates, patients with DLB had a more rapid cognitive decline than ever before.

DLB patients usually have complex pathologies such as cortical Lewy bodies and Alzheimer-type pathology, which may be a reason for a more rapid decline in cognition compared to AD (12). During the pandemic, most of the care of patients with dementia was carried out at home. The clinical profiles of DLB and AD are different, and DLB patients generally have more severe psychiatric symptoms and a higher prevalence of sleep disorders than those with AD. The care of patients with DLB may be more difficult, and a lack of available professional care may accelerate their decline.

That DLB is characterized by more rapid progression than MCI and AD is not only reflected in cognitive function, but also NPS. NPI deteriorated in ~54.5% of the DLB patients, compared to 43.8% of AD and 22.0% of MCI patients during the first year of the COVID-19 pandemic. It is notable that the increase NPI scores (2.7 points) during 1 year was significant in AD, while the change of 2.5 points in DLB was not significant. This could be because the NPI scores were much higher at baseline in DLB compared to AD, so that the NPI increase of 2.5 points in DLB was not as obvious. Despite this our findings are still consistent with previous studies. Researchers conducted a multicenter nation-wide survey Italy and interviewed 4,913 participants with AD, DLB, frontotemporal dementia (FTD) and vascular dementia (VD) at 1 month after the imposition of a quarantine and found that 59.6% patients had worsened behavioral and psychological symptoms of dementia (BPSD) (13). In addition, a study with a small sample size in Spain showed worsening NPI in AD (*n* = 20) and MCI (*n* = 20) by approximately 6 points and 4.5 points after 5 weeks of domiciliary confinement (14).

The collected evidence supports the role of lockdown and confinement in worsening cognition and NPS in patients with MCI and dementia. Further, using multiple linear regression analysis, we found that the decline of social contact was related to the increased NPI scores in AD and DLB and to declining MMSE scores in DLB during the first year of the COVID-19 pandemic. Pre-pandemic studies also suggested that social isolation could predict a steeper decline in cognition and NPS (15–17). Several hypotheses may explain the association between social isolation and cognitive function. Social isolation may lead to augmented stress reactivity that is linked to prolonged activation of the hypothalamic-pituitary-adrenal (HPA) axis and the sympathoadrenal system and to glucocorticoids resistance, which are assumed to have deleterious effects on the prefrontal cortex and the hippocampus (18, 19). At the same time, oxidative stress affects the metabolic and peripheral immunity organs. Thus, the inflammation caused by stress might contribute to other common chronic diseases such stroke or diabetes, which are additional risk factors for cognitive decline (20). Furthermore, long term social isolation can induce loneliness, which is also known to have negative impacts on cognitive function and NPS (21, 22).

PA, which is accepted as a modifiable risk factor in dementia (23), was sharply decreased in all participants during the lockdown, and the of multiple linear regression analysis showed a positive correlation between reduced PA and cognitive decline in AD. PA can improve the management of cardiovascular risk factors such as diabetes, hypertension and obesity, increase neurogenesis and synaptic plasticity and even predict brain function and structure (24).

Considering that previous studies were conducted immediately after 1 to 2 months of lockdown, the differences between our and those studies may be the short-term or long-term impact after lockdown on people with dementia. Few articles discuss this. Compared with the present study, patients tended to have worse NPI scores immediately after lockdown than at 6 months after lockdown. We thought that 6 months were enough for acute stress in dementia patients to be alleviated as they became adjusted to their new lifestyle.

The COVID-19 pandemic has had significant impact on the elderly, and particularly on elderly individuals with AD and related disorders. The epidemic began to worsen again during late 2020, and many countries in the world renewed lockdown policies, which is a new challenge for patients and caregivers. The results of this study suggest that patients should maintain regular exercise and socialization routines during COVID-19 confinement periods by means of home-based workouts that include endurance, resistance and balance exercises or app-based exercise training with online partners (25) and by taking advantage of virtual socialization through technologies such as social media, videoconferencing and internet training (26).

The strength of this study is that it is a longitudinal 1-year follow-up study after a lockdown period during the COVID-19 pandemic. We used a variety of scales to evaluate the global cognitive function and NPS of dementia patients at two time points to reveal the similarities and differences between different dementia types. Furthermore, we quantified PA and social contact and tried to predict risks of worsening cognition and NPS. There are also some limitations in this study. It is a single-center study and the sample size is small. The statistical analysis was limited by this and the present findings must be interpreted with caution. There exists memory bias. This study did not include the healthy control group with the COVID-19 pandemic and lockdown. Confinement and lockdown not only reduce the PA and social contact, but can also introduce other factors such as an unhealthy diet or poor sleep quality, which should be discussed in future research. Also, the cognitive fluctuation in DLB increased the difficulty of the follow-up works. Other related researches about patients and caregiver burdens are also currently underway.

## Conclusion

Patients with MCI, AD and DLB had different progression of cognitive decline and NPS during the first year of the COVID-19 pandemic. Patients with DLB showed rapid worsening after the initial 4-month lockdown in China. Reduced PA and social contact during confinement had a long-term impact on cognition and NPS in dementia patients. During quarantine and stay-at-home mandates, caregivers should help patients with cognitive impairment and dementia to maintain home exercise routines of a certain intensity and frequency and to maintain social contact with friends and relatives by phone and internet.

## Data Availability Statement

The original contributions presented in the study are included in the article/supplementary files, further inquiries can be directed to the corresponding author/s.

## Ethics Statement

The studies involving human participants were reviewed and approved by ethical committee of Tianjin Huanhu Hospital. The patients/participants provided their written informed consent to participate in this study.

## Author Contributions

YJ designed the study. Z-CC, SL, and JG wrote the report. Z-CC and LM did the statistical analyses. XD, HZ, JH, JX, HW, MF, YD, YY, PD, and X-DW contributed to the interpretation and discussion of results and reviewed the manuscript. The collaborating authors contributed to the collection of clinical data. All the authors and the collaborating authors contributed to the article and approved the submitted version.

## Conflict of Interest

The authors declare that the research was conducted in the absence of any commercial or financial relationships that could be construed as a potential conflict of interest.

## Publisher's Note

All claims expressed in this article are solely those of the authors and do not necessarily represent those of their affiliated organizations, or those of the publisher, the editors and the reviewers. Any product that may be evaluated in this article, or claim that may be made by its manufacturer, is not guaranteed or endorsed by the publisher.
